# Efficacy of the local endometrial injury in patients who had previous failed IVF-ICSI outcome

**Published:** 2012-11

**Authors:** Mustafa Kara, Turgut Aydin, Nurettin Turktekin, Karacavus S

**Affiliations:** 1 *Department of Obstetrics and Gynecology, Bozok University, Medical Faculty, Yozgat, Turkey.*; 2 *Department of Obstetrics and Gynecology, Kayseri In vitro Fertilization Center, Kayseri, Turkey.*; 3 *Department of Nuclear Medicine, Bozok University Medical Faculty, Yozgat, Turkey.*

**Keywords:** *Local endometrial injury*, *In vitro fertilization*, *Embryo transfer*, *Pregnancy*

## Abstract

**Background: **The latest studies reported that local endometrial injury is a useful method to improve the success of IVF-ICSI outcome.

**Objective:** To assess whether local endometrial injury occurred by Pipelle in the spontaneous cycle could improve implantation rate, cleavage rate, and pregnancy outcome in the subsequent IVF-ICSI cycle in patients who had recurrent IVF failure.

**Materials and Methods:** An endometrial biopsy was performed on day 21^st^ in 41 patients as intervention group in this retrospective cross-sectional study. The control group contained 42 women.

**Results:** Implantation rate was 22.5% and 10.5% in intervention and control group, respectively and this difference was found to be statistically significant (p=001). Pregnancy rate was 43.9% in the intervention group and this parameter was significantly lower in control group (21.4%) (p=0.03).

**Conclusion:** Local endometrial injury in the nontransfer cycle increases the implantation rate and pregnancy rate in the subsequent IVF-ICSI cycle in patients who had previous failed IVF-ICSI outcome.

## Introduction

IVF-ICSI is performed widely in the world and implantation is essential as much as follicular development for a successful treatment. Especially, implantation is necessary for a successful pregnancy and requires a healthy blastocyst apposition and endometrial receptivity (1, 2). 

Many cytokines (IL-6, IL-8, and TNFα), matrix metalloproteinases (MMP), and molecules characterizing early implantation have been discovered (3, 4). Barash *et al* reported that endometrial biopsy taken spontaneous cycle that preceded IVF improves the IVF-ICSI outcomes such as implantation rate, clinical pregnancy rate, and live birth rate (5). They claimed that the cause of implantation improvement was secondary modifications due to an injury-induced inflammatory reaction. Despite considerable advances in assisted reproductive techniques (ART), management of the patients who had recurrent IVF failure is still a challenge. 

The aim of this study was to detect the efficacy of the endometrial biopsy performed in the nontransfer cycle proceeding the IVF-ICSI cycle. 

## Materials and methods

This is a retrospective cross-sectional study. The patient characteristics (basal hormone levels, duration of infertility, number of attempt, body mass index, antral follicle count, and age) were analyzed, retrospectively. The groups were homogen in terms of these parameters. Inclusion criteria were at least one previous failed IVF-ICSI cycle with a fresh embryo, having good response in the previous IVF cycle, and less than or equal to 37 years old. 

The cases in which testicular sperm extraction (TESE) procedures were performed were not included the study. Spermogram values of the patients were in normal ranges. Male factor patients were excluded from the study. The patients whose body mass index were (BMI) >30 were not included into the study. Standard IVF-ICSI procedure was performed for all of the patients. The patients were randomized to two groups. All women were assessed with baseline day 3 follicle stimulating hormone (FSH), antral follicle count (AFC), estradiole (E_2_) level, and hysteroscopy (H/S) on the 21st day of the previous cycle. Day 21 of the cycle has been assessed by ultrasonography and estradiole (E_2_) level.

The patients in the study group underwent endometrial sampling once, with a biopsy catheter (Pipelle; Gynetics Medical Products, Hamont Achel, Belgium) on the 21^st^ day of the nontransfer cycle. The Pipelle was moved up and down into the uterine cavity. All the women were given Indomethacine 25mg 30-60 minutes prior the procedure. Ciprofloxacin 500 mg was given twice daily for 7 days after the biopsy. Contraception was suggested to women in the nontransfer cycle. 

In long protocol cases, pituitary was down-regulated with Leuprolide acetate (Lucrin ® daily 0.25 mg Abbott, USA). Lucrin was given for at least 10 day starting on the 21^st^ day of the menstrual cycles. On the 2nd day of menstruation, transvaginal ultrasonography (TV USG) and serum E_2_ level were monitorized to show the suppression. Controlled ovarian stimulation (COS) was performed with FSH starting on cycle day 3. Highly purified-urinary FSH (Fostimon HP ® 75 IBSA, Switzerland) was administered. Average FSH starting dose was 450 iu and the dose was individually adjusted according to the previous treatment cycles, body mass index (BMI), and age. 

Pituitary was down-regulated with Cetrorelix (Cetrotide® 0.25 Merck-Serono, Switzerland) in the antagonist protocol starting on the sixth or seventh day of the cycle according to the follicular growth (when the leading follicle reached to 13-14 mm) and E_2_ level (when the level exceeded 600-800 pg/ml). Cetrorelix was continued until the day of hCG. COS was performed with either r FSH or HP-u FSH starting on cycle day 3. Age, body mass index (BMI), and response to the former treatment were used to detect the beginning FSH dose. Average FSH starting dose was 225 IU.

Ovulation induction was monitorized by using E_2_ measurement and TV USG. Human chorionic gonadothropin (Pregnyl® 5000 IU × 2, Schering-Plough, USA) was administered when the dominant follicle reaches 17 mm. Oocytes were retrieved by TV USG- guided needle aspiration. IVF- ICSI was performed in all cases. Embryos were classified according to percentage of fragmentation and blastomere appearances as type I, II, III or IV on 1^st^, 3^rd^ and 5^th^ days. Up to two embryos were transferred into the uterine cavity on day 2, 3 or 5. Luteal phase was supported by progesterone administration (Crinone %8 vaginal gel ® Merck-Serono, Switzerland). Progesterone was given during 12 days after oocyte retrieval. 

Progesteron support was maintained until the 12^th^ gestational week in the pregnancy cases. Implantation failure was defined as the failure of embryo to achieve pregnancy. Fertilization rate was determined as the number of embryos ( >1 cell) divided by the total of oocytes. Cleavage rate was defined as total number of day-3 embryos by total number of fertilized oocytes. Implantation rate was defined as the percentage of embryos given and resulted with pregnancy. Clinical pregnancy was defined by the presence of a fetal sac at ultrasound examination six weeks after embryo transfer. 

**Statistical analysis**

Statistical analysis was performed using SPSS 10.00 (SPSS Inc., Chicago). The Chi-square test was used for categorical variables and an independent sample t-test was used for continuous variables that were normally distributed. p-value<0.05 was considered significant. 

## Results

In total 83 patients were included into the study and only one cycle for each patient was used. Forty one patients were included into the local endometrial injury group, and 42 women were included into the control group. The patient characteristics of both groups were comparable (Table I). The characteristics of the patients were the same. The patients age, duration of infertility, basal FSH levels, basal E_2_ levels, BMI, AFC, and number of previous attempt were assessed but there was no statistical difference. 

Endometrial thickness on hCG day, number of 12-17mm follicles on hCG day and number of >18mm follicles on hCG day, size of dominant follicle, retrieved oocyte number, and transferred embryo number were compared (Table II). All of these parameters were similar and the differences were not statistically significant. The IVF-ICSI outcomes are shown in Table III. The implantation rate was significantly higher in the study group as compared with the controls (22.5% vs. 10.5%, p=0.01). The clinical pregnancy rate was more in the intervention group than control (43.9% vs. 21.4%), and this difference was found to be statistically significant, p=0.03.

**Table I:** Characteristics of the patients

	**Local injury group (n=41)**	**Control group (n=42)**	**p-value**
Age	32.1 ± 5.51	30.1 ± 5.2	0.34
Duration of infertility (year)	7.9 ± 4.8	6.1 ± 3.6	0.09
Number of previous attempt	3.3 ± 1.8	2.5 ± 0.9	0.09
BMI (body mass index)	28.4 ± 5.5	27.2 ± 4.2	0.29
Basal FSH level (iu/l)	6.13 ± 1.2	6.63 ± 1.3	0.84
Basal E_2_ level (pg/ml)	39.4 ± 11.8	41.7 ± 13.6	0.42
AFC (antral follicle count)	9.9 ± 5.2	10.5 ± 5.6	0.66

**Table II:** Comparison of the IVF-ICSI outcomes of the patients performed local injury or not

		**Local injury group (n=41)**	**Control group (n=42)**	**p-value**
Indication				
	Unexplained	18	24	NM
	Male factor	12	7	NM
	Poor reserve	5	4	NM
	Tubal factor	3	2	NM
	Endometriosis	2	1	NM
	Anovulation	1	4	NM
Protocol				
	Antagonist protocol	35	31	NM
	Long protocol	6	11	NM
ET on hCG day (mm)		9.9 ± 1.6	10.6 ± 1.4	0.06
Fol. no. 12-17mm on hCG day		8.4 ± 2.5	9.6 ± 2.8	0.23
Fol. no. >18mm on hCG day		4.2 ± 1.1	5.9 ± 2.0	0.36
Dom. Fol. on hCG day (mm)		20.2 ± 1.4	18. ± 1.2	0.18
Retrieved oocyte no.		10.4 ± 6.3	11.2 ± 7.4	0.61
Transferred embryo no.		1.9 ± 0.5	2.0 ± 0.3	0.49

ET: Endometrial thickness.

NM: Not measured.

**Table III:** Comparison of the IVF-ICSI outcomes in two groups

	**Local injury group (n=41)**	**Control group (n=42)**	**p-value**
Fertilization rate n (%)	274/430 (63.7)	307/493 (62.2)	0.27
Implantation rate n (%)	18/80 (22.5)	9/85 (10.5)	0.01*
Cleavage rate n (%)	258/430 (60)	256/493 (51.9)	0.16
Clinical pregnancy rate n (%)	18/41 (43.9)	9/42 (21.4)	0.03*

* p< 0.05.

## Discussion

Loeb first reported that scratching the guinea-pig uterus during the progestational phase increased the decidualization of the endometrium (6). Decidua formation was simulated by suturing the uterine horn and intra-uterine injection of oil in rodents. These experimental animal studies shown that local injury of the endometrium increases successful implantation percentage (7, 8). Barash *et al* performed endometrial biopsies four times (on the days 8, 12, 21, and 26) during the spontaneous cycle in their prospective case-control study.

All of the 45 patients were good responder and the clinical pregnancy rate, implantation rate, and live birth rate were more than control patients (66.7% vs. 30.3%, 22.7% vs. 14.2%, 48.9% vs. 22.5%, respectively). The efficacy of the endometrial biopsy performed in the nontransfer cycle was confirmed by Raziel *et al* (9). They studied 60 women who had recurrent IVF failure. The Pipelle biopsy was performed twice. They reported a significant increase in the success of IVF-ICSI in the endometrial biopsy group. A more recent study was done by Zhou *et al* in 2008 (10). The endometrial biopsy procedure was performed during the cycle of treatment (on the day 10) unlike the other studies. The outcomes were found to be significantly better in the intervention group. 

Zhou *et al* also investigated the gene expressions. They found some upregulated and downregulated genes. As a conclusion, they suggested that implantation improvement occurred as the result of injury-induced inflammatory reaction. Kalma *et al* reported that endometrial injury taken twice in the spontaneous cycle led to upregulation and downregulation in some genes. Endometrial injury was accepted as a causative factor for these gene modifications (11). 

More recently, a randomized controlled study indicating the success of the local endometrial injury in the IVF-ICSI outcome was done by Narvekar *et al*. They utilized Pipelle to create endometrial injury in 100 women with recurrent implantation failure. Endometrial injury was done twice in the follicular and luteal phase of the nontransfer cycle. Finally, they reported that endometrial injury in the nontransfer cycle increased the percentage of clinical pregnancy, implantation rate, and live birth rate (12).

Despite the unbelievable improvements in ART, embryo implantation remains the rate-limiting step for the success of IVF. We aimed to demonstrate the efficacy of the endometrial injury performed in the spontaneous cycle that preceded the IVF-ICSI. The results of our study were similar with the previous publications. The limitation of our study was the restricted number of patients. Prospective and randomized trials are required to evaluate the influence of local endometrial injury in the IVF-ICSI cycles.
